# Interactive Effects of Nitrogen-Fixing Bacteria Inoculation and Nitrogen Fertilization on Soybean Yield in Unfavorable Edaphoclimatic Environments

**DOI:** 10.1038/s41598-019-52131-7

**Published:** 2019-10-30

**Authors:** Carlos Felipe dos Santos Cordeiro, Fábio Rafael Echer

**Affiliations:** Department of Agronomy, São Paulo Western University, Raposo Tavares HWY, Km 572, 19067-175 Presidente Prudente, São Paulo Brazil

**Keywords:** Drought, Drought, Heat, Heat, Rhizobial symbiosis

## Abstract

The objective of this work was to evaluate the effect of nitrogen (N) fertilization and the biological nitrogen fixation efficiency in soybean crops grown in unfavorable environments (high temperature, low fertility, and areas with sandy soil). Six field experiments were conducted between 2015 and 2018. Two experiments were performed per year. They were conducted in two separate areas. One was previously covered with degraded pasture (post-pasture area). The other was previously used to plant soybean (post-soybean crop area). The treatments consisted of inoculations with N-fixing bacteria (NFB) (0, 4, 8, and 12 doses ha^−1^) and N fertilization with rates of 0, 25, 50, and 100 kg ha^−1^. N fertilization and inoculation with NFB increased soil N, though the levels were still low. Among the tested groups, those with the application of eight doses of inoculant recorded the highest grain yields in post-soybean areas. They showed 10% (237 kg ha^−1^) and 15% (336 kg ha^−1^) higher grain yields when compared to crops treated without inoculant and crops with four doses of inoculant, respectively. N fertilization with 25 and 100 kg ha^−1^ decreased the root nodules of soybean plants grown in the post-soybean and post-pasture areas, respectively. Soybean crops grown on degraded pasture areas also showed good response to N fertilization (50 kg ha^−1^) when combined with NFB inoculation (12 doses ha^−1^). These showed grain yields 22% (439 kg ha^−1^) higher than those of plants treated with just 12 doses of inoculant and no N fertilization.

## Introduction

Soybean (*Glicine max* L. Merr) is one of the world’s main agricultural crops. Soybean grains are a source of oil and protein. They are widely used in food and feed products. The world soybean production in the 2017/2018 crop season was approximately 346.9 million Mg. The largest producers were the United States, Brazil, and Argentina, accounting for 82% of total world production^1^. Brazil is the second largest producer, with an area of 35.1 million hectares (ha) and an average grain yield of 3.4 Mg ha^−1^. It is the main soybean producer in tropical climate regions^2^.

Soybean cultivation in marginal areas are increasing in recent years due to restrictions for the opening of new areas and increasing demand for soybean oil and bran. These areas are often covered by degraded pastures and have sandy soil, with low water retention capacity, low phosphorus content, low organic matter, and a low capacity for the supply of nitrogen to plants^3,4^. Pastures cover approximately 175 million ha in Brazil; approximately 80% of this area (140 million ha) is completely degraded or presents some degree of degradation^5^. Approximately 74.5 million ha are on sandy or light textured soil distributed in different biomes, mainly in the Cerrado region^6^.

Nitrogen (N) is the most required nutrient for soybean plants, and the export of N per grain harvest is estimated as 50 kg of N per Mg of grain. This N is mainly supplied via biological nitrogen fixation (BNF) (80%), soil organic matter, and eventually nitrogen fertilizers that are applied during sowing (20%). BNF represents an economy of $15 billion dollars for Brazilian producers^7^.

Abiotic stress (from high temperatures, water deficits, low soil fertility, and soil acidity) provides obstacles to BNF and N supply to plants^8–10^. This entails the need for N fertilization to increase soybean grain yield. Scharf and Wiebold^11^ reported a response of soybean plants to N application (33 kg ha^−1^) in soils with a pH lower than 6 and nitrate contents (0–60 cm soil layer) lower than 56 kg ha^−1^. Caliskan *et al*.^12^ found an increase in soybean grain yield with N application of up to 80 kg ha^−1^ in soil with a pH above 7 and high CaCO_3_ levels. Ray *et al*.^13^ reported an increase in soybean grain yields of 7.7% and 15.5% with the application of 320 kg ha^−1^ of N in irrigated and non-irrigated environments, respectively. They attributed the improved response in non-irrigated areas to the sensitivity of BNF to water deficit stress.

The average world temperature is predicted to increase up to 0.4 °C in the next two decades, which would likely increase drought events in some regions^14^. These increases would be more pronounced in tropical regions, leading to the faster drying of soil in these areas^15^. This may increase agricultural loss due to water and thermal stress. In such conditions, BNF efficiency may be reduced due to the sensitivity of these microorganisms to drought^16^ and thermal stresses^10^.

Most scientific studies report that N fertilization has no effect on soybean grain yield. This is due to the loss in root nodulation efficiency that causes the plants to be dependent on mineral fertilizer, increasing production costs^17,18^. However, according to Menza *et al*.^19^, soybean plants with high grain yields respond to N application. The climate of the region and the adopted production system may affect the response of soybeans to N fertilization^20^. Hungria *et al*.^21^ used an inoculant with a high concentration of rhizobia. They reported low root nodulation in areas of new soybean cultivation. Pavanelli and Araújo^22^ found lower soybean root nodulation in areas with low phosphorus contents. Therefore, soybean crops grown in agricultural systems where yield is restricted (due to low soil fertility, water deficits, and high temperatures) may respond well to the application of N.

The hypothesis of this work was that N fertilization may increase soybean grain yield in the first year in areas with sandy soil previously covered by degraded pasture. Thus, our objective was to evaluate the response of soybean plants to the inoculation with NFB in interaction with nitrogen fertilization in post-degraded pasture and post-soybean areas.

## Results

### Yield and production components

**Figure 1:**
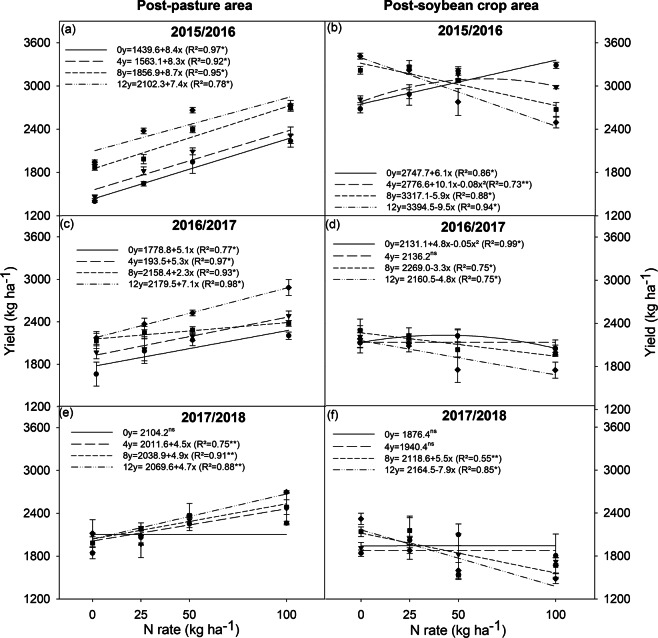
Yield of soybean plants grown in areas previously covered by degraded pasture (post-pasture area) and a soybean crop (post-soybean crop area), under different inoculant doses (0, 4, 8, and 12) and nitrogen rates (0, 25, 50, and 100 kg ha^−1^), in the 2015/2016, 2016/2017, and 2017/2018 crop seasons. *significant at 1%, and **significant at 5%; ns = not significant.

**Figure 2:**
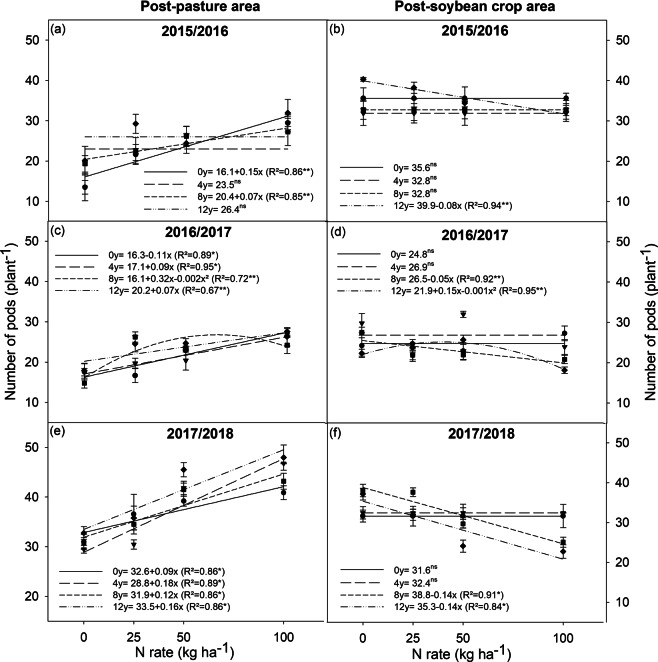
Number of pods per plant of soybean grown in areas previously covered by degraded pasture (post-pasture area) and a soybean crop (post-soybean crop area), under different inoculant doses (0, 4, 8, and 12) and nitrogen rates (0, 25, 50, and 100 kg ha^−1^), in the 2015/2016, 2016/2017, and 2017/2018 crop seasons. *significant at 1%, and **significant at 5%; ns = not significant.

During the three years of research, the grain yield of post-pasture soybean crops showed a linear response that increased when inoculant doses and N fertilization rates increased, except for the inoculant dose of zero in the 2017/2018 crop season (Fig. 1a,c,e). This increase was attributed to the higher number of pods per plant (Fig. 2), as there was no effect on the number of grains per pod and on grain weight. This occurred despite that the application of N caused a linear reduction in grain weight (average of the three seasons) (Table 1). In addition, the application of 12 doses of the inoculant (twice the number of recommended doses in new soybean areas) was advantageous for the post-pasture soybean crops in two of the three studied years (2016/2017 and 2017/2018). Post-soybean crops recorded the highest grain yields with eight and 12 doses of inoculant, and showed an overall grain yield reduction when applying N (except for dose zero and four, in all years) (Fig. 1b). This was due to the decreased number of pods per plant (Fig. 2) and grain weight (Table 1). The effect of N rates on the number of grains per pod was not conclusive.

**Table 1:** Nitrogen leaf content, number of grains per pod, and 1000-grain weight of soybean plants grown in areas previously covered by degraded pasture (post-pasture area) and a soybean crop (post-soybean crop area), in the 2015/2016, 2016/2017, and 2017/2018 crop seasons.

Crop season (S)	Number of grains		1000-grain weight		N-leaf concentration	
	pod^−1^		g		g kg^−1^	
	Post-pasture area	Post-soybean crop area	Post-pasture area	Post-soybean crop area	Post-pasture area	Post-soybean crop area
2015/2016	2.12 b	2.24 b	134.2 a	126.9 a	36.9 a	53.1 c
2016/2017	2.76 a	2.45 a	122.5 c	98.1 c	31.3 c	63.6 a
2017/2018	2.07 b	2.43 a	126.7 b	107.2 b	34.1 b	58.4 b
**Inoculant dose (I) – doses ha**^**−1**^						
0	2.29	2.48	126.8	112.8	32.1	57.5
4	2.34	2.37	128.0	111.5	33.3	57.7
8	2.28	2.30	129.9	112.3	35.3	59.0
12	2.35	2.39	127.1	109.2	35.7	59.3
Y	2.38^ns^	2.5 + 0.04x + 0.003x²R² = 0.97**	127.3^ns^	111.5^ns^	32.2 + 0.31x R² = 0.84*	57.4 + 0.17x R² = 0.93*
**Nitrogen rate (N) – kg ha**^**−1**^						
0	2.35	2.29	132.1	114.1	27.7	57.1
25	2.30	2.47	129.5	109.8	32.1	57.8
50	2.34	2.34	126.6	111.9	35.2	58.3
100	2.27	2.45	123.7	109.1	41.3	60.3
Y	2.31^ns^	2.38^ns^	13.1 − 0.018x R² = 0.94*	11.2 − 0.004x R² = 0.60**	28.2 + 0.13x R² = 0.99*	57.1 + 0.03x R² = 0.98*
**F**						
S	0.0000	0.0000	0.0000	0.0000	0.0000	0.0000
N	0.5738	0.0530	0.4420	0.2031	0.0000	0.0001
I	0.4206	0.0223	0.0003	0.0073	0.0000	0.0000
S*N	0.0614	0.1072	0.0000	0.0863	0.0441	0.6720
S*I	0.9478	0.5431	0.5681	0.6762	0.0978	0.4921
N*I	0.5320	0.8269	0.6150	0.1953	0.0000	0.0000
S*N*I	0.3969	0.8973	0.4590	0.3069	0.0269	0.8655
CV%	11.4	13.1	8.1	6.8	9.4	3.8

*Significant at 1%, **significant at 5%, ns = not significant. Means of the treatments followed by the same letter do not differ by Tukey’s test at 5% probability a > b (*p* < 0.05).

### N-leaf concentration

**Figure 3:**
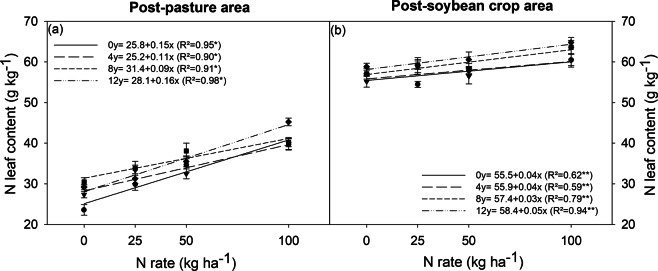
N leaf content of soybean plants grown in areas previously covered by degraded pasture (post-pasture area) and a soybean crop (post-soybean crop area), under different inoculant doses (0, 4, 8, and 12) and nitrogen rates (0, 25, 50, and 100 kg ha^−1^). *significant at 1%, and **significant at 5%; ns = not significant.

**Figure 4:**
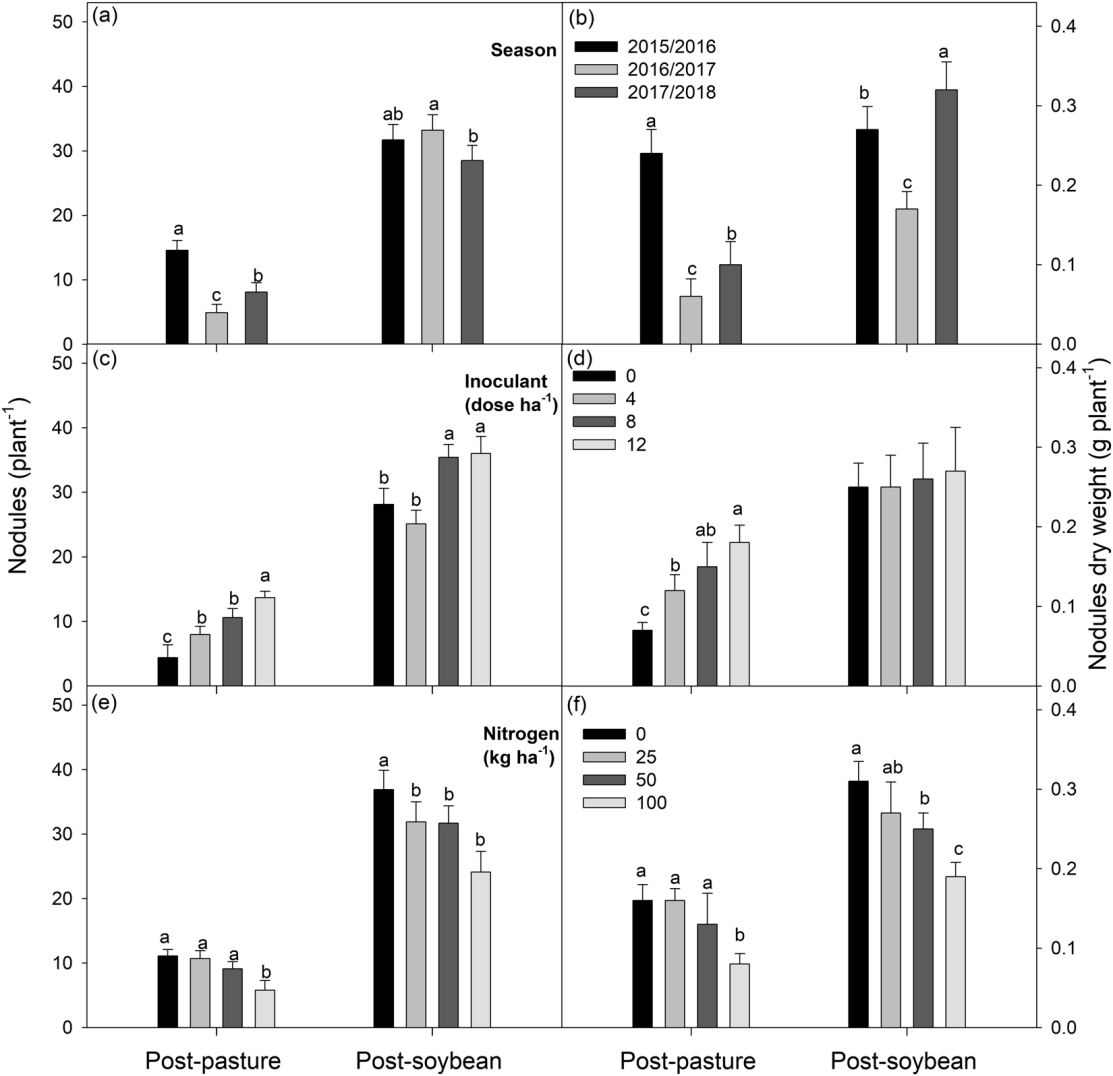
Number and dry weight of root nodules of soybean plants grown in areas previously covered by degraded pasture (post-pasture area) and a soybean crop (post-soybean crop area), in the 2015/2016, 2016/2017, 2017/2018 crop seasons (**a,b**), according to the inoculant doses, expressed by specific season and N rates (**c,d**); according to the application of N fertilizer, and expressed by specific season and inoculant dose (**e,f**). Means of the treatments followed by the same letter do not differ by the Tukey’s test at 5% probability (*p* < 0.05).

The N-leaf concentration of soybean plants in post-pasture areas increased linearly by increasing inoculant doses and N rates (Fig. 3, Table 1). The highest N-leaf concentration was found in the 2015/2016 crop season, when the plants exhibited the highest root nodulation (Fig. 4a,b). The leaf nitrogen concentration of post-soybean plants was in the range of sufficiency or slightly above it, showing little effect from the inoculant doses and N rates (Fig. 3; Table 1).

### Root nodulation

The number and weight of root nodules in plants grown in the post-pasture area and post-soybean crop area were affected by the crop season due to water deficits (Figs S1, S2). The average number of root nodules was, in general, lower in post-pasture plants (9.23) than in post-soybean plants (31) (Figs 4a,b). The number and weight of root nodules of soybean plants grown in the post-pasture area showed a linear response with increased inoculant doses, reaching 15 and 0.18 g, respectively, with 12 doses of inoculant. The use of eight doses of inoculant in post-soybean plants was sufficient to reach high root nodulation (36 root nodules per plant) (Fig. 4c). However, the inoculant doses did not affect root nodule weight (Fig. 4d). The application of 100 kg ha^−1^ of N reduced the number and weight of root nodules of post-pasture plants. The application of 25, 50, and 100 kg ha^−1^ of N reduced the number and weight of root nodules, respectively, in post-soybean plants (Fig. 4e,f).

**Figure 5:**
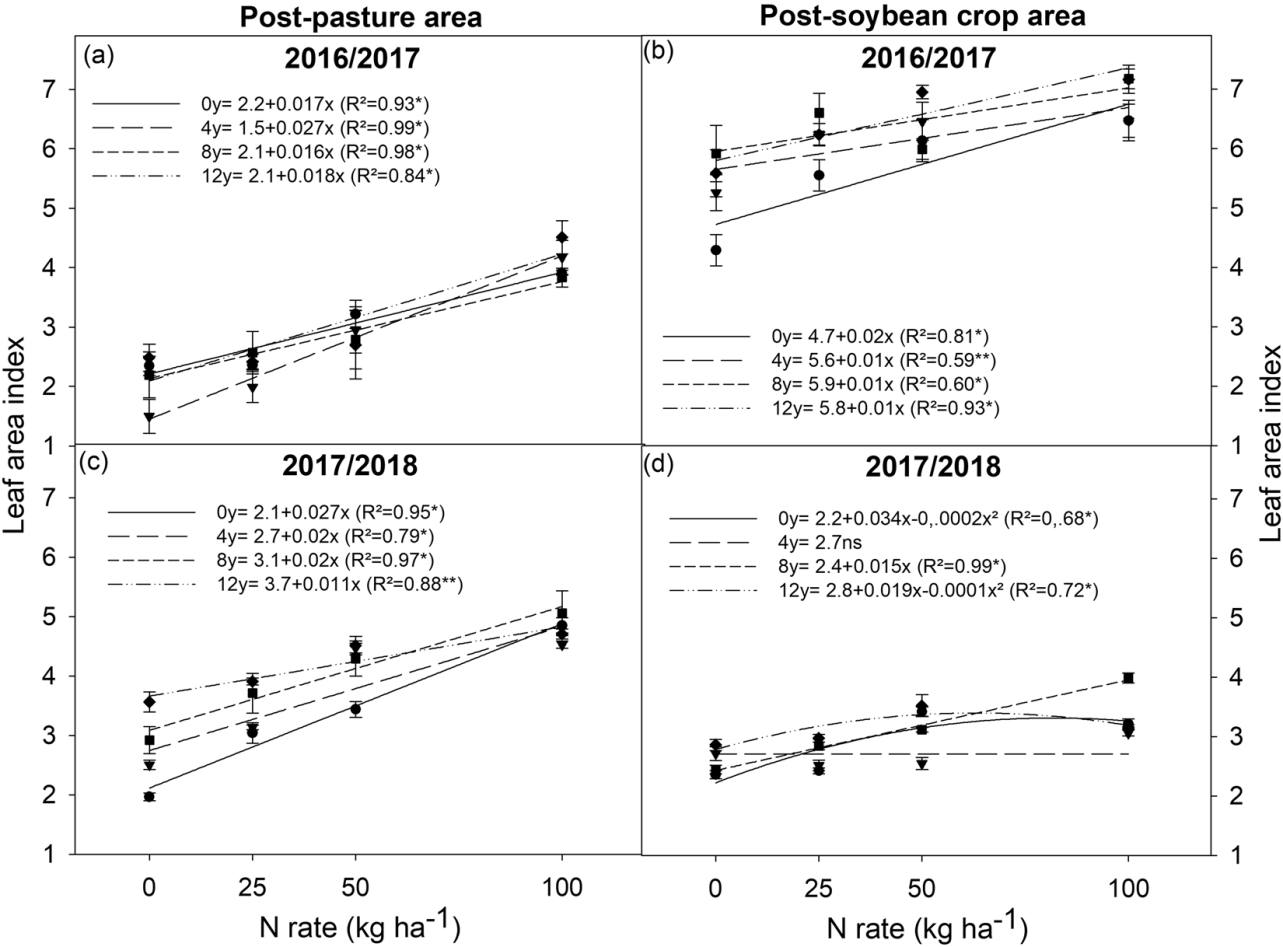
Effect of nitrogen rates (0, 25, 50, and 100 kg ha^−1^) within each inoculant dose (0, 4, 8, and 12) on leaf area index of soybean plants (R4 stage) grown in areas previously covered by degraded pasture (post-pasture area) and a soybean crop (post-soybean crop area), in the 2016/2017 and 2017/2018 crop seasons. *significant at 1%; **significant at 5%; ns = not significant.

### Leaf area index

Nitrogen fertilization in the post-pasture area linearly increased the leaf area index (LAI) of soybean plants in the two evaluated years (Fig. 5a,c). In the absence of N, LAI was significantly affected by the inoculant doses in the 2017/2018 crop season (Fig. 5c), but no effect was observed in 2016/2017. The difference in LAI between inoculant doses was more pronounced at low N rates (0 and 25 kg ha^−1^). It declined with increasing N rates and showed no difference when using 100 kg ha^−1^ of N. Soybean plants grown in the post-pasture area did not show an LAI higher than five in any crop season (Fig. 5). Soybean plants grown in the post-soybean crop area in the 2016/2017 crop season showed an LAI of 4.3 (without a dose of inoculant and no N) to 7.16 (12 doses of inoculant and 100 kg ha^−1^ of N), but there was no effect of N when combined with the other doses of inoculant (Fig. 5b). LAI was similar between treatments (N rates and inoculant doses) in the 2017/2018 crop season. It reached 3.99 with eight doses of inoculant and 100 kg ha^−1^ of N.

### Soil nitrogen

**Figure 6:**
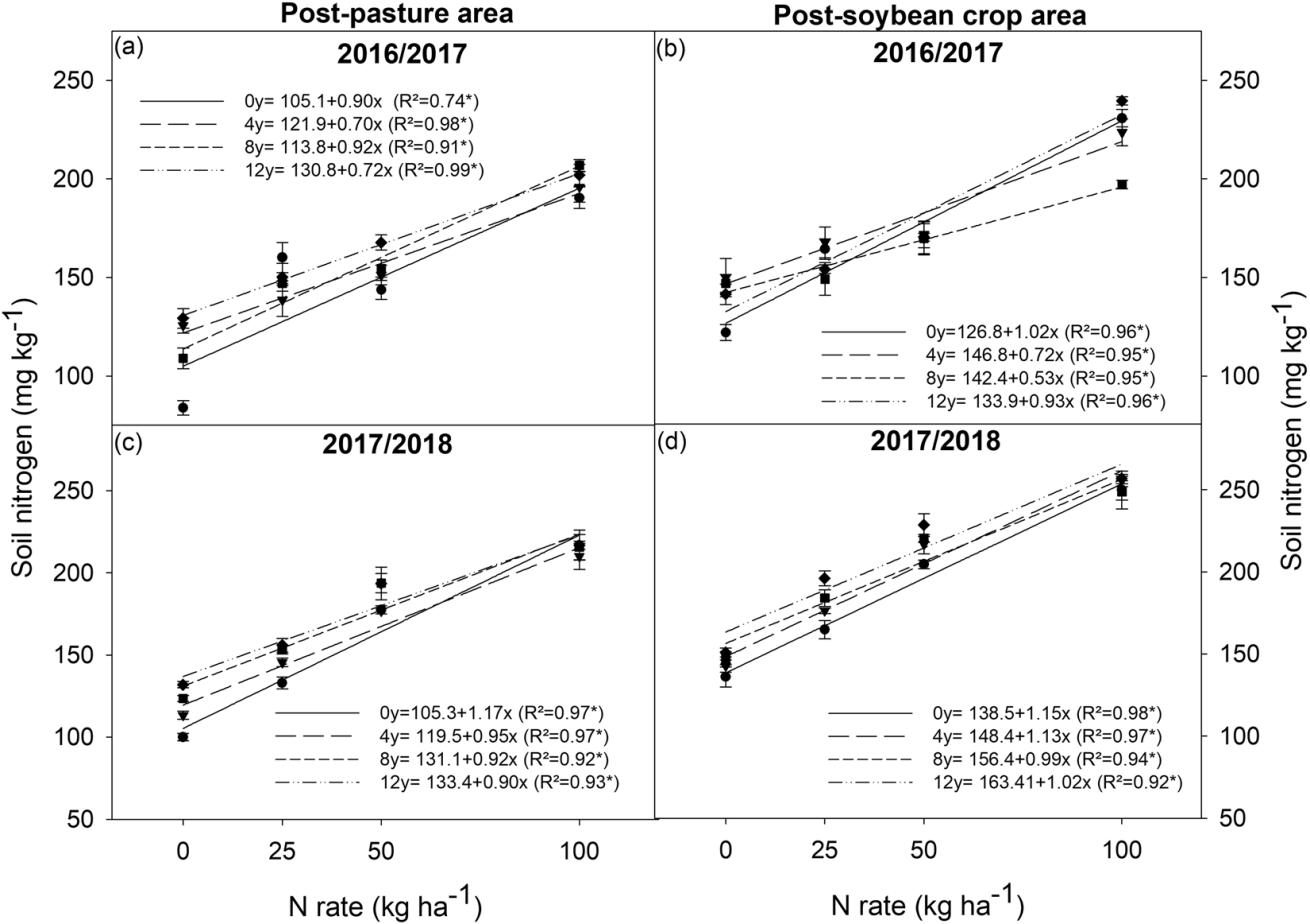
Soil nitrogen concentration in post-pasture and post-soybean crop areas in the 2016/2017 and 2017/2018 soybean crop seasons. *significant at 1%; **significant at 5%; ns = not significant.

Soil N concentration increased by increasing the N rates and inoculant doses (Fig. 6), especially under lower N rates (0 and 25 kg ha^−1^) in the post-pasture area (Fig. 6a,c). Soil N content did not change much when increasing inoculant doses in the post-soybean crop area (Fig. 6b,d).

The number of root nodules, weight of root nodules, and leaf N concentration of soybean plants in the post-pasture and post-soybean crop areas did not show much correlation with grain yield. Soil N concentration and LAI of soybean plants in the post-soybean crop area showed low rates of correlation with the grain yield. However, the soil N content and the LAI of plants in the post-pasture area had a significant correlation with grain yield in all evaluated crop seasons (Table 2).

**Table 2:** Pearson correlation coefficient between grain yield and number of root nodules, nodule dry weight, leaf nitrogen content, soil nitrogen content, and leaf area index.

Season	Nodules#		Nodule dry weight		Leaf N content		Soil N content		Leaf area index	
	Post-pasture area	Post-soybean crop area	Post-pasture area	Post-soybean crop area	Post-pasture area	Post-soybean crop area	Post-pasture area	Post-soybean crop area	Post-pasture area	Post-soybean crop area
2015/2016	0.11^ns^	0.24^ns^	0.06^ns^	0.29**	0.68*	0.009^ns^	—	—	—	—
2016/2017	0.29**	0.27**	0.19^ns^	0.35*	0.64*	−0.33*	0.69*	−0.48*	0.56*	−0.48*
2017/2018	−0.20^ns^	0.25**	−0.42*	0.53*	0.66*	−0.26**	0.63*	−0.57*	0.62*	−0.57*

*significant at 1%; **significant at 5%; ns = not significant.

**Figure 7:**
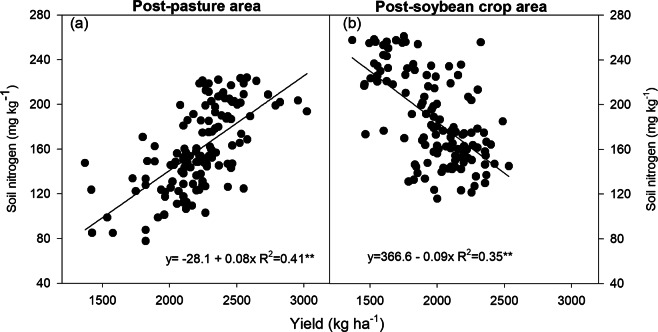
Correlation between grain yield and soil nitrogen concentration of soybean plants grown in areas previously covered by degraded pasture (post-pasture area) and a soybean crop (post-soybean crop area) (average two crops season). *significant at 1%; **significant at 5%; ns = not significant.

**Figure 8:**
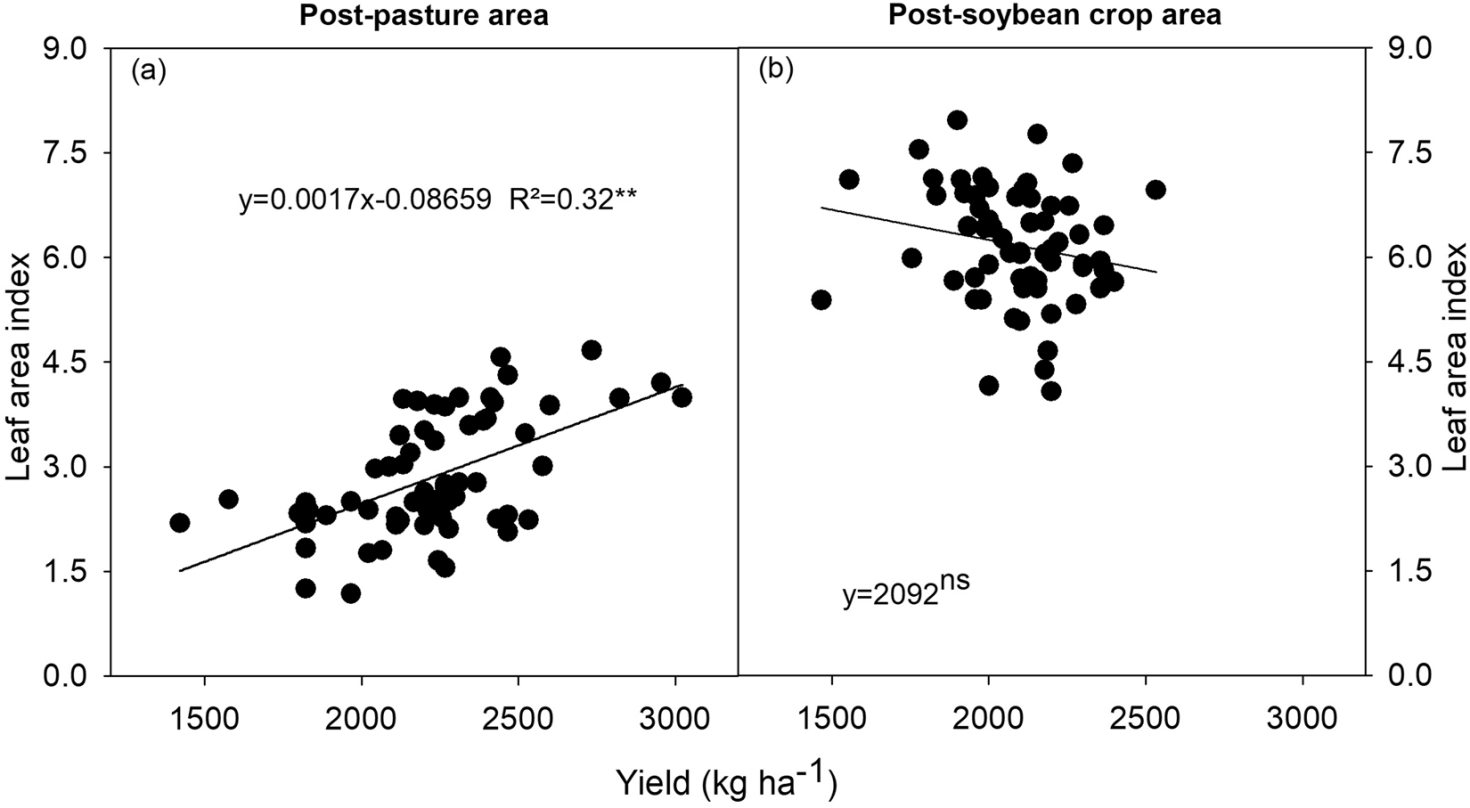
Correlation between grain yield and leaf area index of soybean plants grown in areas previously covered by degraded pasture (post-pasture area) and a soybean crop (post-soybean crop area). *significant at 1%; **significant at 5%; ns = not significant.

The higher the N content in the soil (Fig. 7a) and the LAI (Fig. 8a), the greater the grain yields of post-pasture plants. The increase in soil N content in the post-soybean area decreased the soybean grain yield (Fig. 7b), while the LAI had no effect on grain yield (Fig. 8b).

## Discussion

Nitrogen fertilization had no effect on soybean plants in the post-soybean crop area when using at least eight doses of inoculant in areas of past soybean growth, as reported by Hungria *et al*.^21^. However, it is worth noting that the water deficit at the R5 stage limited the soybean grain yield in the 2016/2017 and 2017/2018 crop seasons (Figs S1, S2). In addition, water deficits^13^, high temperatures^10^, acidic soil^8^, and low fertility soil (especially soil in areas with phosphorus contents lower than 10 mg dm^−3^)^21,22^ may inhibit root nodulation.

The use of 12 doses of inoculant (100% higher than the recommended dose) did not result in the highest soybean grain yield among the tested post-pasture plants in the absence of N. The highest grain yields were found in the treatment with 12 doses of inoculant and 100 kg ha^−1^ of N, denoting that the edaphoclimatic conditions of the experimental site were not favorable to biological nitrogen fixation (BNF) (Fig. S1, Table S1). However, the economical and non-detrimental N rate to BNF for the post-pasture plants was 50 kg ha^−1^ of N (Fig. 4e,f), with a 22% increase in grain yield (439 kg ha^−1^) when compared to the treatment with 12 doses of inoculant and no N fertilization (average of the three crop seasons).

Most studies have shown no response in terms of soybean grain yield with N when BNF is efficient^23–27^. Some studies have shown that N increases soybean grain yields, but without economic viability^13,17,28^. However, soybean crops with high grain yields may respond well to N fertilization^19^, depending on the production system and climate of the region^20^. Environments that limit BNF (as those found in the present work) may result in gains in soybean grain yields through the use of N fertilization in the first year. This is due to the low soil fertility (Table S1), an absence of initial inoculum of N-fixing bacteria, high N demands for mineralization of crop residues (Table S3), and the N content in the soil (Fig. 6) that limits the availability of N to plants. Considering the expansion of soybean areas in Brazil over degraded pastures and areas with sandy soil, the annual variation in grain yield due to climatic factors (from 26% to 34%) may increase. This is from the increased risk of production failure in these environments, as reported for maize and rice. These showed annual grain yield variations of 0.9 and 0.5 Mg ha^−1^, respectively^29^.

Depending on the edaphoclimatic conditions, nitrogen-fixing bacteria (NFB) can persist in the soil for up to ten years^24^. This is a disadvantage for new areas, in which root nodulation can range from 5 to 25 root nodules per plant. This occurs even when using high doses of inoculant^21^. This was observed in the present study, as the use of 12 doses ha^−1^ of inoculant resulted in 13 root nodules per plant (Fig. 4). Without these doses, the root nodulation rates for soybean plants grown in the post-soybean area were adequate. This was observed through the improvement of soil fertility (Table S1), an increase in soil N content (Fig. 6), and the presence of *Bradyrhizobium* in the soil due to inoculation of the previous crop. Even without inoculation, the plants exhibited more than 28 nodules (Fig. 4). Similarly, Hungria *et al*.^21^ found plants with 40 root nodules in areas with previous soybean crops and without inoculation with NFB.

Nitrogen fertilization with 25 and 100 kg ha^−1^ of N reduced root nodulation of soybean plants in the post-soybean crop area and post-pasture area, respectively (Fig. 4). This may be due to N availability in the soil, as soybeans can leave 30 to 40 kg ha^−1^ of N in the soil after harvesting^7^. This association with nitrogen fertilization reduced the root nodulation in the post-soybean area. The increase in soil N content reduces soybean root nodulation^9,26,27^. However, low N rates have no effect or benefit to root nodulation^30,31^. This was found in the post-pasture area of the present study, where the application of up to 50 kg ha^−1^ of N did not affect root nodulation (Fig. 4).

In the post-pasture area, it was necessary to apply 100 kg ha^−1^ of N and 12 doses ha^−1^ of inoculant to achieve N-leaf concentration above 45 g kg^−1^. Moretti *et al*.^32^ obtained soybean grain yields above 4 Mg ha^−1^ with N-leaf concentrations above 45 g kg^−1^. This produced a grain yield of 3.5 Mg ha^−1^ with leaf N content of 39 g kg^−1^ at the R4 stage^33^. The N leaf concentrations of post-soybean plants were greater than 50 g kg^−1^, denoting the efficiency of the BNF.

An adequate leaf area index (LAI) allows for the interception of radiation and conversion of light into chemical energy (carbohydrates), resulting in high soybean grain yields (5 to 6 Mg ha^−1^)^34^. The optimum LAI is 6 to 6.5 in the R5 stage^34^. However, the optimal LAI for soybean crops of lower productive potential, from 3 to 4 Mg ha^−1^, as those in the current study is 3.5 to 4^35^. The LAI of post-soybean plants in the 2016/2017 crop season varied between 4.5 and 7.1. This decreased grain yield (Fig. 1d) and grain weight (Table 1) were due to the 15-day water deficit at the grain filling stage (Fig. S1b). Therefore, as expected, crops with a high LAI growing in areas with sandy soil with low capacity for water retention create favorable conditions for dry soils due to increased plant transpiration^36^.

## Conclusions

The grain yield of soybean plants grown in post-pasture areas can increase despite unfavorable edaphoclimatic environments through the application of moderate N rates combined with high inoculant doses. Inoculation and N fertilization in soybeans grown in post degraded pastures would be recommended in areas with sandy soil. However, the inoculation only would be necessary in the post-soybean areas.

## Material and Methods

### Characterization of the experimental area

Six field experiments were conducted between 2015 and 2018 in Presidente Bernardes in the state of São Paulo, Brazil (22°11′53″S, 51°40′30″W, and with an altitude of 401 m). The climate of this region is classified as Aw (Köppen). The rainfall and maximum/minimum temperatures during the experiments are shown in Fig. S1. The soil water content during the 2016/2017 and 2017/2018 crop seasons are shown in Fig. S2. The soil of the areas was classified as Ultisol (of sandy texture)^37^. The results of the physical and chemical soil analyses are shown in Table S1.

### Experimental setup

The experiment was conducted in a randomized block experimental design with four replications, with inoculum (NFB) doses (0, 4, 8, and 12 doses ha^−1^) in the plots and nitrogen rates of 0, 25, 50, and 100 kg ha^−1^ in the subplots. The experimental units were of 7.00 × 3.15 m, totaling 22.05 m^2^.

Two experiments were conducted simultaneously in the crop seasons. We used an area previously covered with degraded pasture with *Urochloa brizantha* cv. Marandu (post-pasture area) and an area previously covered with a summer soybean crop, which was then fallowed and covered by *U. brizantha* due to the soil seed bank (post-soybean crop area). The *U. brizantha* cv. Marandu plants were dissecated 40 days before sowing. This was performed using a glyphosate-based herbicide (3 L ha^−1^ of the commercial product). Liming and fertilization were performed following the recommendations of Sousa and Lobato^38^, according to the soil analysis (Table S1). Liming was performed 30 days before the soybean sowing (TMG 7062 IPRO cultivar), and potassium was applied as a topdressing 30 days after emergence. Seeding was carried out with a spacing of 0.45 m between the rows. Information on liming and fertilizer rates, sowing time, and plant density are shown in Table S2.

NFB was inoculated in the soil (in the sowing furrows) using *Bradyrhizobium japonicum* (SEMIA 5079 and SEMIA 5080) at 6 × 10^9^ CFU ml^−1^ (one dose was equal to 100 ml). The solution was applied with a flow rate of 50 L ha^−1^. Leaf applications of cobalt (8 g ha^−1^) and molybdenum (40 g ha^−1^) were performed at the V3 stage of the soybean plants. Nitrogen (ammonium nitrate 33% N) was applied manually at the R1 stage to treatments with N fertilization. Herbicide, insecticide, and fungicide applications were carried out according to the recommendations for the crop.

The remaining *U. brizantha* cv. Marandu biomasses were evaluated before the soybean sowing in areas of 0.4 × 0.5 m. This was performed through the collection of straws from the soil. Soil samples of 2000 cm^3^ (0.1 × 0.2 × 0.2 m) were collected to evaluate the root biomass. The roots were washed, dried in an oven at 65 °C for 48 hours, weighed, and grounded for carbon and nitrogen analysis^39^ (Table S3).

### Sampling and data collection

#### Root nodulation

Root nodulation (number and weight of root nodules) was evaluated at the R4 stage in six plants per plot. The root nodules were counted manually, dried in an oven at 65 °C for 48 hours, and weighed on a precision scale (0.01 g) to determine their dry weight.

#### Leaf nitrogen content

Leaf nitrogen content was evaluated at the R4 stage of the soybean plants in ten trifoliate leaves (without petioles) per plot^39^.

#### Leaf area index (LAI)

LAI was evaluated at the R4 stage (55 days after emergence) using a ceptometer (AccuPAR LP-80^®^; Decagon Devices).

#### Soil nitrogen content (total-N)

Soil nitrogen content was evaluated at the R4 stage according to the methodologies described by Cantarella and Trivelin^40^. Soil samples of the 0–0.20 m layer were collected from each experimental area, with three sub-samples for each plot. The samples were stored in hermetically sealed containers containing liquid nitrogen until evaluation. Total N was determined by digestion with quantitative N-nitrate recovery. The moisture of all samples was corrected to perform the calculations. Only the 2016/2017 and 2017/2018 crop seasons were evaluated.

#### Yield and production components of soybean

The yield components (stand, number of pods per plant, number of grains per pod, and 1000-grain weight) and grain yields were determined at the R8 stage. One meter of each row was collected to evaluate the yield components. The mechanical harvesting of the three central rows (7 m each) was performed to evaluate grain yield. Grain moisture was corrected to 13%.

#### Statistical analysis

The statistical analysis consisted of analysis of variance and regression analysis. Means were compared by Tukey’s test at a 5% probability level. Graphs were plotted using Sigmaplot®.

## Supplementary information

INTERACTIVE EFFECTS OF NITROGEN-FIXING BACTERIA INOCULATION AND NITROGEN FERTILIZATION ON SOYBEAN YIELD IN UNFAVORABLE EDAPHOCLIMATIC ENVIRONMENTS

## Data Availability

The datasets generated during and/or analyzed during the current study are available from the corresponding author upon reasonable request.
