# Publisher Correction: Interactive Effects of Nitrogen-Fixing Bacteria Inoculation and Nitrogen Fertilization on Soybean Yield in Unfavorable Edaphoclimatic Environments

**DOI:** 10.1038/s41598-020-69171-z

**Published:** 2020-07-21

**Authors:** Carlos Felipe dos Santos Cordeiro, Fábio Rafael Echer

**Affiliations:** Department of Agronomy, São Paulo Western University, Raposo Tavares HWY, Km 572, 19067-175 Presidente Prudente, São Paulo Brazil

Correction to: *Scientific Reports* https://doi.org/10.1038/s41598-019-52131-7, published online 30 October 2019

In the original version of this Article, the author Carlos Felipe dos Santos Cordeiro was incorrectly indexed. This error has now been corrected.

