# Supplementary material for: Interactive Effects of Nitrogen-Fixing Bacteria Inoculation and Nitrogen Fertilization on Soybean Yield in Unfavorable Edaphoclimatic Environments
Source: Sci Rep. 2019 Oct 30;9:15606. doi: 10.1038/s41598-019-52131-7 (PMC6821774; doi:10.1038/s41598-019-52131-7)
Supplement: Supplementary file 1 — INTERACTIVE EFFECTS OF NITROGEN-FIXING BACTERIA INOCULATION AND NITROGEN FERTILIZATION ON SOYBEAN YIELD IN UNFAVORABLE EDAPHOCLIMATIC ENVIRONMENTS [file 41598_2019_52131_MOESM1_ESM.pdf]

INTERACTIVE EFFECTS OF NITROGEN-FIXING BACTERIA INOCULATION AND NITROGEN FERTILIZATION ON SOYBEAN YIELD IN UNFAVORABLE EDAPHOCLIMATIC ENVIRONMENTS

*Carlos Felipe dos Santos Cordeiro[1] & Fábio Rafael Echer[1]

[1]Department of Agronomy, São Paulo Western University. Raposo Tavares HWY, Km 572, 19067-175, Presidente Prudente, São Paulo, Brazil. *Corresponding author: cordeirocfs@gmail.com

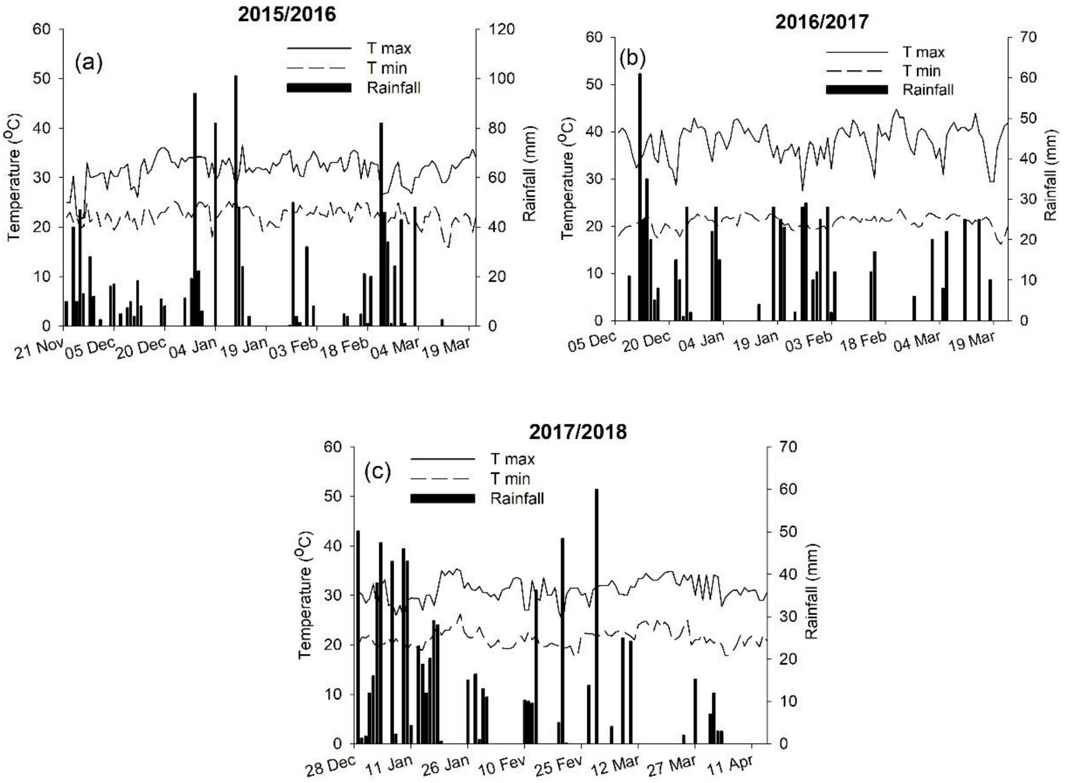

Figure supplementary S1. Rainfall, and maximum and minimum temperatures during the experiments, in the 2015/2016, 2016/2017, and 2017/2018 crop seasons. Presidente Bernardes, São Paulo, Brazil.

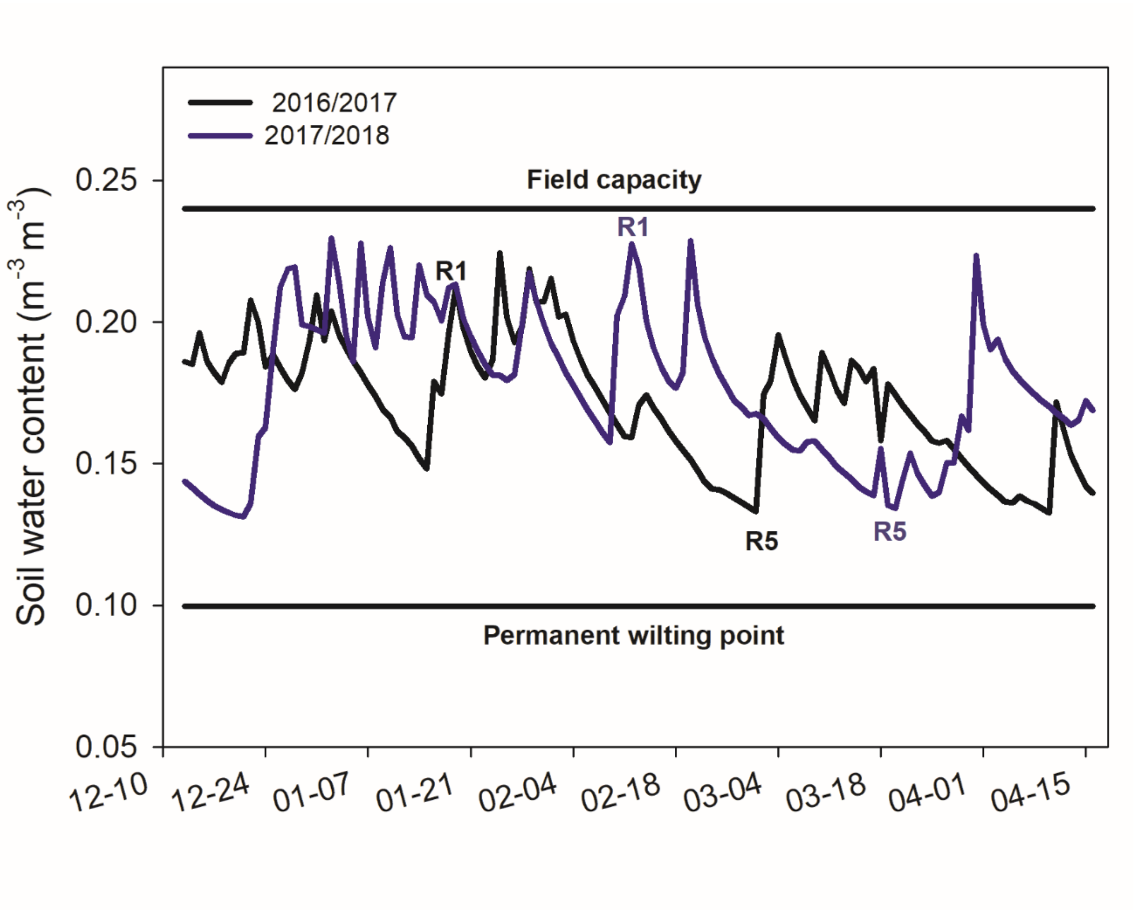

Figure supplementary S2. Soil water volume (m^3) in the 0.0-0.5 m soil layer during the experiments, in the 2016/2017 and 2017/2018 crop seasons.

Crop season	pH	SOM	P	H+Al	K	Ca	Mg	CEC	Sand	Clay	Soil density
		g dm^{-3}	mg dm^{-3}	..........................mmol dm^{-3}......................					g kg^{-1}		g cm^{-3}
Post-pasture area											
2015/2016	5.0	8.9	2.1	15.9	0.6	7.5	3.8	27.5	844	101	--
2016/2017	4.3	16.2	0.1	24.2	0.6	1.6	1.4	27.7	830	105	1.61
2017/2018	5.0	10.9	5.0	17.3	0.7	6.1	3.7	27.8	824	106	1.62
Post-soybean crop area											
2015/2016	5.2	13.8	12.9	19.7	1.8	16.2	11.1	48.8	830	121	--
2016/2017	5.0	12.8	11.8	17.6	0.6	11.8	5.9	35.9	852	110	1.67
2017/2018	4.8	10.3	20.3	22.9	1.3	12.0	2.9	39.2	852	100	1.61

Table supplementary S1. Soil physical and chemical properties (0-0.20 m soil layer) of the post-pasture area and post-soybean crop area, in the 2015/2016, 2016/2017, and 2017/2018 soybean crop seasons.

	2015/2016		2016/2017		2017/2018	
	**Post-pasture area**	**Post-soybean crop area**	**Post-pasture area**	**Post-soybean crop area**	**Post-pasture area**	**Post-soybean crop area**
Liming (kg ha^{-1})	1400	900	1800	850	2500	1800
($N-P_2O_5-K_2O$) (kg ha^{-1})	17-129-73	21-75-43	20-150-50	21-75-43	16-120-40	20-70-40
K_2O Topdressing (kg ha^{-1})	60					
Sowing time	21/11/2015		05/12/2016		28/12/2017	
Density plant (m^{-2})	31.1	30.9	29.5	33.4	22.9	23.8

Table supplementary S2. Liming and fertilizer rates, sowing time, and density of soybean plants in the 2015/2016, 2016/2017, and 2017/2018 crop seasons.

**Crop season**	**Post-pasture area**		**Post-soybean crop area**	
	**Dry matter**	**Carbon/nitrogen**	**Dry matter**	**Carbon/nitrogen**
	**kg ha^{-1}**	**-**	**kg ha^{-1}**	**-**
	Shoot			
2015/2016	1592	50.4	1992	44.9
2016/2017	1615	69.8	2266	38.3
2017/2018	1188	47.7	5574	56.1
	Root			
	kg m^{-3} (kg ha^{-1})	C/N	kg m^{-3} (kg ha^{-1})	g kg^{-1}
2015/2016	1.0 (2000)	90.5	0.9 (1990)	60.8
2016/2017	1.3 (2760)	112.6	1.12 (2240)	97.8
2017/2018	0.4 (855)	52.7	1.62 (3240)	65.8

Table supplementary S3. Dry matter and carbon to nitrogen ratio of the previous crop (*Urochloa brizantha*) to soybean.
